# Robust Detection of Critical Events in the Context of Railway Security Based on Multimodal Sensor Data Fusion

**DOI:** 10.3390/s24134118

**Published:** 2024-06-25

**Authors:** Michael Hubner, Kilian Wohlleben, Martin Litzenberger, Stephan Veigl, Andreas Opitz, Stefan Grebien, Franz Graf, Andreas Haderer, Susanne Rechbauer, Sebastian Poltschak

**Affiliations:** 1 AIT Austrian Institute of Technology, 1210 Vienna, Austria; kilian.wohlleben@ait.ac.at (K.W.); martin.litzenberger@ait.ac.at (M.L.); stephan.veigl@ait.ac.at (S.V.); andreas.opitz@ait.ac.at (A.O.); 2 Joanneum Research Forschungsgeselllschaft mbH, 8010 Graz, Austria; stefan.grebien@joanneum.at (S.G.); franz.graf@joanneum.at (F.G.); 3 Joby Austria GmbH, 4040 Linz, Austria; andreas.haderer@joby.aero (A.H.); susanne.rechbauer@joby.aero (S.R.); sebastian.poltschak@joby.aero (S.P.)

**Keywords:** sensor data fusion, multi-sensor fusion, surveillance of critical infrastructure

## Abstract

Effective security surveillance is crucial in the railway sector to prevent security incidents, including vandalism, trespassing, and sabotage. This paper discusses the challenges of maintaining seamless surveillance over extensive railway infrastructure, considering both technological advances and the growing risks posed by terrorist attacks. Based on previous research, this paper discusses the limitations of current surveillance methods, particularly in managing information overload and false alarms that result from integrating multiple sensor technologies. To address these issues, we propose a new fusion model that utilises Probabilistic Occupancy Maps (POMs) and Bayesian fusion techniques. The fusion model is evaluated on a comprehensive dataset comprising three use cases with a total of eight real life critical scenarios. We show that, with this model, the detection accuracy can be increased while simultaneously reducing the false alarms in railway security surveillance systems. This way, our approach aims to enhance situational awareness and reduce false alarms, thereby improving the effectiveness of railway security measures.

## 1. Introduction

Even with today’s advanced sensor and surveillance technologies, ensuring seamless security surveillance in the railway sector is a significant challenge. Railway infrastructure consists of large assets with a wide geographical spread. Permanent monitoring and surveillance of the open track and its assets to prevent security incidents such as vandalism, trespassing, sabotage and even equipment theft are technically challenging. However, seamless monitoring and surveillance of the railway infrastructure form the basis of any security strategy.

A review of the literature reveals that permanent threats to the safety and integrity of critical railroad infrastructure have been extensively studied, with numerous articles published on the subject, including, for example, [1,2,3]. Killen et al. conducted a comprehensive analysis of six studies on the social background and motivation behind railroad-related graffiti vandalism, as well as the technological and non-technological mitigation measures employed. In one of the studies, the authors presented a deep-learning-based methodology for robust graffiti detection, as reported in [4]. While CCTV surveillance and geographical information systems (GISs) are employed to record and identify geographical patterns in vandalism activities, it is also observed that the presence of surveillance can have unintended consequences. For instance, individuals may be deterred from engaging in vandalism at locations where surveillance is installed and instead relocate their activities to other locations. This highlights the need for near-seamless surveillance of the infrastructure to counteract such a relocation effect.

In their study, Grabusic et al. examine the social and demographic factors associated with railway trespassing accidents. They conclude that fencing is the most effective method for preventing trespass, but that it is impractical and costly to implement in all areas along a railway line. In their review of technological solutions, CCTV combined with automatic person detection by computer vision using convolutional neural networks (CNNs), as well as LiDAR (light detection and ranging), are presented. However, the authors conclude that further trials and tests are required to assess the realistic impact of these technological solutions. In their survey of technological solutions for automated surveillance for trespassing prevention, Zhang et al. cite the work of [3] and provide an in-depth analysis of video analytics based on CCTV using CNNs. They also examine multi-sensor fusion with thermal camera technology and sound and vibration sensors. In their conclusion, the necessity of preventing security operators from being overwhelmed by the information overload from a multitude of sensor systems is explicitly pointed out, while sensor fusion is suggested as a possible mitigation method.

The increasing risk of terrorist attacks on critical infrastructure has made railways particularly vulnerable, due to their relatively unguarded nature in open track sections in sparsely inhabited areas. The manipulation and placing of objects on the track with malicious intent represents a new quality of threat beyond vandalism.

As the size and geographic extent of the surveillance system increase, the difficulty of conducting surveillance efforts also rises. This is due to the fact that the amount of surveillance data and the information produced by the surveillance equipment both scale in proportion to the system’s size. The technical limitations of specific sensor types in detecting certain threats necessitate the integration of diverse sensor technologies to reach a comprehensive situational awareness. However, without the deployment of further post-processing strategies, an increased number of sensors will inevitably lead to an increased overall rate of false alarms. At a certain point, this may even lead to information overload for the security operators. A critical issue encountered in the context of false alarms is that they not only exhaust the resources that the security operators have to spend on their investigation, but they also severely undermine confidence in the surveillance system. In the worst case, correct alarms will not be handled anymore by operators, rendering the surveillance system useless.

A number of recent papers have attempted to address this problem [5,6,7,8]. However, their approaches typically rely on a single sensor technology combined with enhanced analytics. Cao et al. implemented video analytics based on a combination of a background model and object classification for trespass detection and benchmarked various classifiers on a test dataset of 15,000 railway trespassing scenes. The performance of the algorithm yielded 96.9% accuracy with a false positive rate of 0.24%. Haryono and Hidayat demonstrated the implementation of real-time GPU-based video analytics based on the “YOLO” image classifier and a tracking algorithm to determine the individuals in a crowd trespassing a predefined line. They reported an accuracy of 84% when surveying a crowd of people in a realistic railway station scenario. An example of the use of multiple sensors, namely millimeter-wave RADAR and LiDAR, was reported in [9] for the detection of foreign objects on a rail track. The detection of both sensors was fused on a spatial grid of 2 m ×1.3 m cell size. The achieved target detection rates were 99.7% with a false positive rate of 0.1%. Although, these results are highly satisfactory, it is important to be aware of the significant impact of false alarms and their importance for practical large-scale surveillance. For example, a false positive rate as low as 0.2% will result in one false alarm per second in a surveillance system covering 500 cameras and running with one frame per second.

This study presents a novel fusion model that facilitates Probabilistic Occupancy Maps (POMs) and Bayesian Fusion as an inference model. The efficacy of this model in enhancing accuracy and reducing false alarms in the context of railway security is demonstrated. This article commences with an overview of related work, with a particular focus on general fusion architectures and the methodologies underpinning our fusion model. A brief summary of related applications of the aforementioned methodology is also provided. In the section entitled ‘Materials and Methods’, we offer a concise description of the sensors and sensor models employed. In the same section, we present the methodologies of our fusion model. This section concludes with a detailed account of the recorded data and test environment, which serve as the foundation for the evaluation of our fusion model, which is described in the later part of this section. In the Results section, the benefits of applying our fusion model to single sensor fusion and multi-sensor fusion are demonstrated. In the ‘Discussion’ section, the findings are presented in terms of the reduction in false alarms. Furthermore, we have identified potential shortcomings and future challenges, which are also discussed in this section.

## 2. Related Work

The utilisation of multiple sensor technologies presents a number of challenges, including the necessity to align the spatio-temporal data produced by the various sensors. This is a complex process that requires the integration of data from multiple sources. Data fusion can be applied at different stages of the signal processing pipeline. While there are various classifications of fusion, it is generally useful to distinguish between signal-level, feature-level and decision-level fusion. This distinction is discussed in detail in the works of [10,11]. Signal-level fusion is typically employed between analogous sensor modalities (e.g., disparate microphones for de-noising), whereas feature- and decision-level fusion may be applied to disparate sensor types with the objective of enhancing the overall performance of a sensor system with respect to accuracy and the reduction in false alarms.

One example of the application of data fusion of different sensors for public surveillance is provided in [12]. The authors utilised a combination of audio and video sensors for the detection of security-relevant events in public areas. Another recent example of a similar system developed with NATO is provided in [13]. Both examples represent instances of feature-level fusion, where the first is concerned with the identification and refinement of the static location of an incident, while the second applies fusion to the tracking of subjects.

### Probabilistic Occupancy Grid Mapping and Bayesian Fusion

Occupancy grid mapping is one of the most popular approaches for geographical mapping. Its usage is prominent in the domain of autonomous driving [14,15,16,17,18]. Mapping multiple sensors’ information such as LiDAR, Radar and cameras to the surroundings of the vehicle in real time for the purpose of autonomous driving is a current topic. Furthermore, occupancy grid mapping is also widely used in the field of robotics, e.g., for path planning [19,20,21] and navigation [22,23]. In combination with the Bayesian filter [23,24,25], they form a robust fusion methodology for challenging use cases in both of those sectors. Nevertheless, they are not limited to these. The authors in [12] also describe a similar approach based on weighted maps in the field of detection of security critical events. They describe that this approach also can be successfully used in a static environment where the occupancy of the map changes based on moving targets such as persons in the vicinity of the sensors.

## 3. Materials and Methods

This section presents the fundamental elements required for the study. It commences with an overview of the sensors and detectors employed, followed by a description of the fusion model. The recorded data are then outlined, and the section concludes with an explanation of the evaluation methodology. We would like to mention at this point, that the main focus of this article is to show the improvement and effectiveness by applying our fusion model in the context of railway security. For this reason, the sensing part, which is described in the beginning, is presented in a way to foster completeness and reproducibility of the work. There is no focus on improving the individual sensing capabilities.

### 3.1. Sensors and Detectors

#### 3.1.1. Person Detector Based on Thermal Imaging

Object detection techniques based on deep learning (DL) can be divided into two general categories: two-stage detectors (e.g., R-CNN, Faster R-CNN) and one-stage detectors (such as YOLO and SSD). Two-stage detectors (proposal generation, then classification) employ two stages to detect objects from an image, and these detectors often provide state-of-the-art (SOTA) results or high accuracy on available datasets. However, these detectors have a lower inference speed compared to one-stage detectors. One-stage detectors are predominantly employed in real-time object detection applications, offering comparable results at a considerably faster pace than two-stage detectors [26].

**Figure 1:**
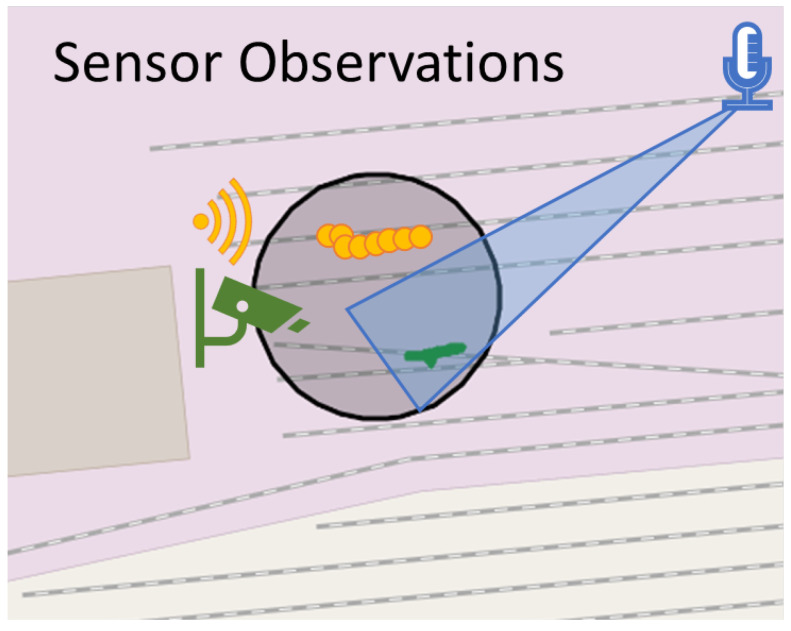
Example of sensor observations and ground truth. Orange small circles—radar observations; green small polygons—thermal observations; blue big polygon—acoustic observation; black big circle—ground truth observation.

The You Only Look Once (YOLOv5) DNN-based detector has gained considerable traction for its real-time capabilities and effectiveness in detecting diverse object classes, as evidenced by its adoption in numerous applications [27]. In this study, the pre-trained YOLOv5 on the dataset COCO was utilized for the detection of individuals at a distance (up to 150 m) using thermal imaging cameras with an image resolution of 640 × 480. Thermal images present unique challenges due to lower quality, reduced contrast, fewer discernible features and increased noise compared to RGB images. To address these challenges, the same model was applied to images captured by two thermal cameras with different focal lengths (wide angle and telephoto) and overlapping fields of view. This allowed the system to cover both short and long distances with appropriate image resolution. As a result, bounding boxes of detected persons were provided by the detector. To provide a location for the fusion model, the bounding boxes were projected on the ground based on the extrinsic parameters (position and orientation) of the cameras utilising a standard pinhole model. As a result, the bounding boxes yielded polygons as seen in Figure 1. The softmax scores of the YOLO were used to provide an estimate for the confidence, which is necessary in the fusion model.

#### 3.1.2. Acoustic Detector Based on an Microphone Array

**Figure 2:**
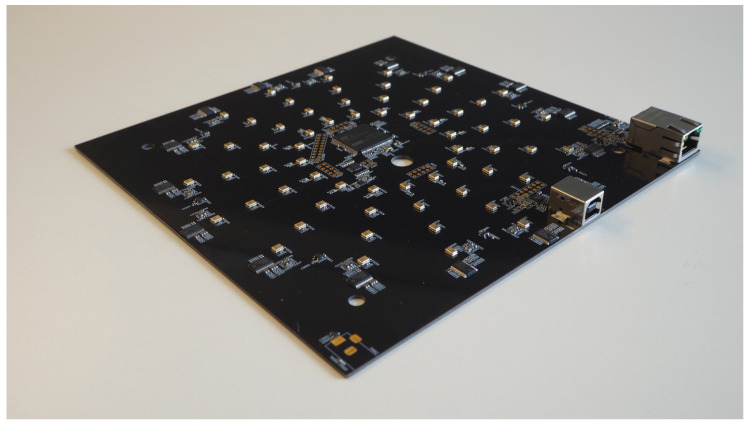
Picture of the 64-ary MEMS array.

Each of the acoustic sensors is equipped with a 64-ary microphone array, depicted in Figure 2, a 9-degree-of-freedom orientation sensor, a global navigation satellite system module, a single-board-computer, a battery and an LTE modem for communication. The signal from a single microphone is used as input for a detection stage to classify the incoming audio with a convolutional recurrent neural network [28] that has been trained with data from a previous measurement campaign. The data used for training were recorded in a similar setup at a different location prior to the recording of this dataset. The training dataset was recorded in a way to generate the same characteristics as needed for this work (speech, rattling and vandalism). It consists of approximately 11 h of data. This ensures that the training data do not bleed into the validation of the system. The neural network uses an 80-band log-mel spectrogram as input and consists of three convolutional and one recurrent layer. The last layer uses a sigmoid activation function to output the probability of a detection.

If a predefined event is detected, e.g., spraying with a graffiti can, the signals of the 64 MEMS microphones are fed to an angle-of-arrival estimator. The microphones are arranged as four concentric circles on a horizontal plane with diameters of {7.2,10.5,13.7,17} cm, respectively. To enable real-time processing, a Bartlett beamformer is employed. This beamformer generates five angle-of-arrival estimates every 2 ms that are fed to a k-means algorithm for clustering and variance estimation. The maximum of the Bartlett spectrum corresponds to the direction of the signal with the most power. Hence, the direction of the maximum is not necessarily equal to the direction of the event. Especially if interfering signals are present, it is paramount to include more than the highest maximum of the Bartlett spectrum. The k-means algorithm generates three angle-of-arrival clusters including its mean and variance in a local reference frame every 2 s. To infer which of these three clusters corresponds to the direction of the detected event, we generate the beamformed signals according to the mean values of the three clusters. These three beamformed signals are then re-classified with the detector, and the direction of the signal with the highest probability is chosen. For the propagation of the detection to the fusion model, the location and probability are essential. We choose to use triangular polygons to describe possible positions of a detected event. We use the mean and variance to describe the direction and opening angle of the triangle, respectively. However, the acoustic measurements do not allow us to estimate the distance of the detected event from the acoustic sensor. Thus, we set the maximum distance to 30 m and scale the triangle polygon accordingly (see Figure 1). The probability for the fusion model is given by the output of the neural network.

#### 3.1.3. Movement Detector Based on Radar Technology

**Figure 3:**
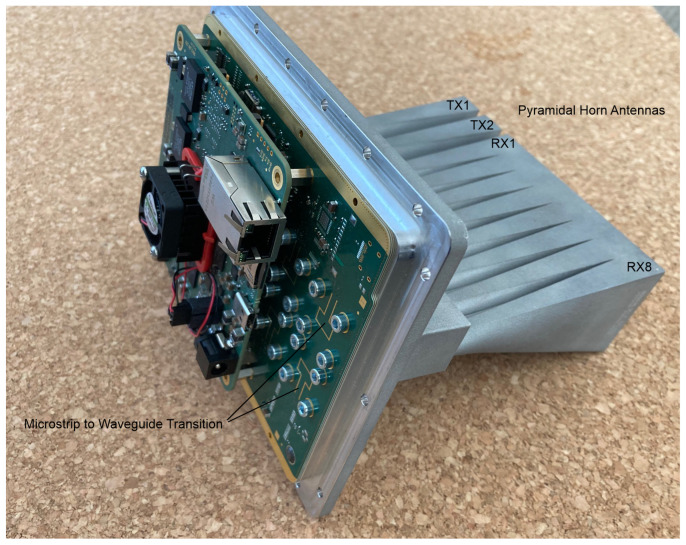
24 GHz radar sensor with 3D-printed pyramidal horn antennas.

Highly integrated automotive transceivers enable the design of powerful, low-cost mm-wave radar sensors. The implemented 24 GHz surveillance radar features two transmit and eight receive channels directly feeding microstrip to waveguide transitions, as shown in Figure 3. The chosen RF buildup supports novel 3D-printed antenna arrays, and therefore, the radiation characteristics can be selected to match the desired field-of-view (FoV). An array with pyramidal horn antennas was used to conduct the measurements.

The antenna positions form a uniform linear array with 16 virtual elements spaced by half the free-space wavelength. Polyphase modulation enables simultaneous activation of both transmit channels. The radar sensor applies range-Doppler processing to evaluate the distance and the radial velocity to targets in the illuminated scene. A third Fourier transform estimates the direction of arrival (DoA) to localize the targets. A detection is generated when the received energy of a bin in the data cube exceeds the threshold derived from the estimated power spectral density (PSD). In a further processing step, the detections are grouped into clusters and assigned to tracks. Finally, a constant velocity Kalman filter outputs the two-dimensional position and velocity at 10 Hz. The Radar System was operated in the ISM band between 24.0 and 24.2 GHZ. In the configuration described, the radar sensor can detect persons up to 350 m. For the fusion model, the location of the detection was modelled as a geo-circle with 1 metre radius (see Figure 1).

### 3.2. Fusion Model—A Bayesian Approach

Our approach is based on the methodologies described in [29]. In this work, we adapt the approach based on occupancy grid mapping, Bayesian Updating and Bayesian Fusion for the purpose of geographical mapping of critical events in the domain of railway security. In contrast to automotive applications, we assume that the region of interest (surveyed area) changes due to continuous sensor observations (e.g., person detection, movement, spraying) reported in the act of a person committing sabotage, vandalism or trespassing. In the following sections, we will present the selected methodology and its derivation from the methodology described in [29].

#### 3.2.1. Probabilistic Occupancy Maps—POMs

The spatio-temporal mapping of sensor observations is based on the use of POMs. A POM is defined as follows:

(1) $$m=\{m_{ij},1\leq i\leq N_{H},1\leq j\leq N_{W}\},$$

where $N_{H}$ and $N_{W}$ denotes the number of rows and columns of the map that represents the region of interest. Thus, $m_{ij}$ represents one cell of a POM. We denote $z_{t}^{k}$ as a sensor observation of the *k*-th sensors observed at the time *t*. For each cell $m_{ij}$, the posterior probability of the cell occupancy is defined as follows:

(2) $$p\left(m_{ij}|z_{t}^{k}\right)\in \left(0,1\right),\;1\leq k\leq K$$

where *K* is the total number of sensors in use. Thus, each cell $m_{ij}$ holds the probability of occupancy estimated by the received sensor observation at a specific time *t*. A probability near 0 means that the occurrence of a critical event is highly unlikely and vice versa. If no sensor information exists, the actual state of a cell is *unknown*. This is also how a map m is initialized if we assume that no prior information about the region of interest is available. The *unknown* state is characterized as follows:

(3) $$p\left(m_{ij}\right)=\frac{1}{2},\;\forall i,j.$$

#### 3.2.2. Bayesian Updating

As sensor observations are continuously generated, it is necessary to update POMs over time. To this end, we employ an updating process that combines Bayesian inference in log-odds form with an exponential decay to model the effect of sensor information aging over time. This approach allows us to account for the diminishing impact of older events. The Bayesian formula in log odds is employed to estimate the posterior probability $p(m_{ij}|z_{1:t_{n}}^{k})$, which infers all sensor observations from prior updating steps 1 to $t_{n}$. The log-odds ratio $l_{t_{n}}^{k}\left(m_{ij}\right)$ at a cell $m_{ij}$ is defined as follows:

(4) $$\begin{array}{cc} l_{t_{n}}^{k}\left(m_{ij}\right) & =\log\frac{p(m_{ij}|z_{1:t_{n}}^{k})}{1-p(m_{ij}|z_{1:t_{n}}^{k})}\\ \; & =l_{t_{n-1}}^{k}\left(m_{ij}\right)\cdot e^{-\frac{\Delta t}{\tau }}+\log\frac{p(m_{ij}|z_{t_{n}}^{k})}{1-p(m_{ij}|z_{t_{n}}^{k})}-\log\frac{p(m_{ij})}{1-p(m_{ij})} \end{array}$$

The following three terms are involved in Equation (4): 1. The previous state of the map, $l_{t_{n-1}}^{k}\left(m_{ij}\right)$, which is reduced by the ’forgetting factor’ described in [29]. 2. The log-odds ratio of the probability distribution, $p(m_{ij}|z_{t_{n}}^{k})$. This represents the probability of each cell in the map, given the current sensor observation, $z_{t_{n}}^{k}$. This is the step where the current estimate of the map is updated with a new sensor observation. Finally, the third term represents the *prior probability* of the map, which will normally be $p(m_{ij})=0.5$ since the map is *unknown* a priori. In the event that prior information regarding the map is available (for instance, blind spots where an observation is physically impossible), it can be incorporated via the *prior probability* of the map. In this work, no prior information about the map was assumed; therefore, (3) holds.

#### 3.2.3. Forgetting Factor

Since in our approach, we decided to use the log-odds form (4), we introduced the forgetting factor in the log-odds set:

(5) $$l_{t_{n}}^{k}\left(m_{ij}\right)\leftarrow l_{t_{n}}^{k}\left(m_{ij}\right)\cdot e^{-\frac{\Delta t}{\tau }},\;\Delta t=t_{n}-t_{n-1}$$

keeping the same characteristics:

(6) $$\lim_{l_{t_{n}}^{k}\left(m_{ij}\right)\to 0}\frac{1}{1+\exp^{-l_{t_{n}}^{k}\left(m_{ij}\right)}}=\frac{1}{2}$$

Thus, we ensure that the decay converges to the *unknown* state. This decay is applied before each updating process. The decay factor τ plays an important role in modelling the impact of past information. It usually is estimated empirically. As a rule of thumb, in this work, it is parameterised proportional to the frequency of the sensor observations provided, e.g., $\tau =\frac{1}{20}$ in case sensor observations from the video detector are sent at 20 Hz. In this work, we used τ=1s uniformly for all sets of configurations.

#### 3.2.4. Bayesian Fusion

In order to integrate all available sensor observations, $z_{t_{n}}^{k}$, multiple maps are defined and fused according to Bayes’ theorem. This approach is analogous to the updating process previously defined. However, there is one restriction that needs to be applied. In the updating process, the priors of the map $p(m_{ij})$ are used to infer information about the map. For the fusion process, this information is omitted so that it will not be inferred multiple times. Consequently, the assumption of (3) is made when calculating the fusion of all maps using (4). The resulting formula for the fusion of k sensors in each cell $m_{ij}$ is then defined as followed:

(7) $$l_{t_{n}}^{1:K}\left(m_{ij}\right)=\sum_{k=0}^{K}l_{t_{n}}^{k}\left(m_{ij}\right)$$

To transform back into the probabilistic form, we use the following function:

(8) $$p\left(m_{ij}|z_{1:t_{n}}^{1:k}\right)=\frac{1}{1+\exp^{-l_{t_{n}}^{1:K}\left(m_{ij}\right)}}$$

#### 3.2.5. Decision

Finally, a decision is made to trigger an alarm if a certain threshold κ∈[0,1] is exceeded for an individual cell (i,j)∈m. The alarm resulting from the decision process is localized at the cells of the resulting map after the fusion process, where the threshold κ is exceeded. This set of cells is denoted as

(9) $$\left\{\left(i,j\right)\in m:p\left(m_{ij}\right)\left(t\right)>\kappa \right\}$$

As each cell $m_{ij}$ represents a location in space (region of interest), a geo-localised alarm is generated. Adjusting the parameter κ allows us to parameterise the sensitivity of the fusion model. This essentially gives us a measure of how much information is needed to trigger an alarm. In our work, we choose this threshold based on end-user requirements, setting it to κ=0.75. It is important to note that this influences the sensitivity of the fusion model. Consequently, the performance evaluation is also affected. In practice, a compromise must be reached when selecting the parameters of the fusion model.

### 3.3. Data Description

**Figure 4:**
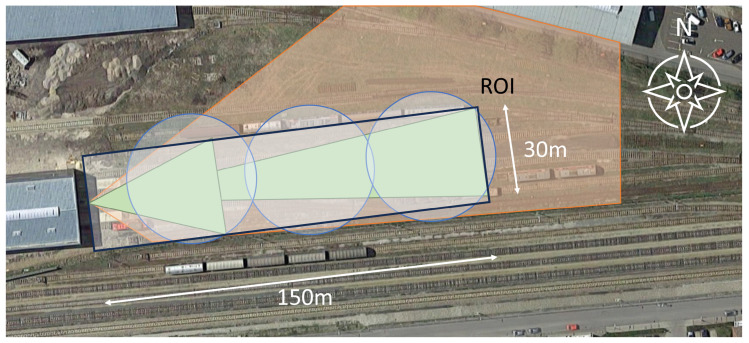
Sensor setup, field of views (FoV) and region of interest (ROI). Green—thermal cameras; blue—acoustic sensor; orange—radar sensor; black—region of interest.

The data were collected in the vicinity of a railway depot in Austria. The region of interest consists of three parallel tracks (30 m) over a length of about 150 m (including old parked wagons—for graffiti). Figure 4 shows the region of interest and the railway depot.

The size of the POM we used in this work for modelling the region of interest was chosen as $\left(N_{H},N_{W}\right)=\left(500,500\right)$, resulting in a spatial resolution of (0.06,0.3) m per pixel. The sensor network consisted of two thermal cameras mounted on a mast 7.5 m above the ground, a radar at a height of 7 m and 3 acoustic sensors placed at ground level. The sensors were placed to take advantage of complementary sensor observations (e.g., person detection + spray can rattling). This resulted in an overlapping detection area of the thermal, radar and acoustic sensors. The placement and field of view (FoV) of the thermal cameras can be seen in Figure 4—in green. For the first thermal camera, a short focal length and wide FoV for the near-field optic was used to obtain a better resolution close to the mast. The second camera used a long focal length far field optic for detection at greater distances (100–150 m). The radar sensor was mounted in line with the direction of the cameras (see Figure 4—orange area). The acoustic sensor placement was chosen to be even across the length of the region of interest (see Figure 4—blue circles). This arrangement was specifically chosen to provide maximum coverage of the region of interest, taking into account the complementary sensor data.

#### 3.3.1. Scenario Description

The present study focused on three use cases that exemplify critical incidents in the domain of railway security.

1. Sabotage—Items placed on railway tracks deliberately by humans (e.g., backpack or metal objects)
2. Trespassing—Usually when entering the area of the railway depot. Or simply when using a shortcut over the rails.
3. Vandalism —Caused by graffiti or deliberately destroying property (e.g., breaking windows of the wagons).

For each use case, the scenarios with the greatest security impact were selected. In addition, a playbook was written for each scenario, describing the modus operandi. All scenarios were performed by the same actors four times in total over a period of daytime (2 cycles), twilight (1 cycle) and nighttime (1 cycle) according to the script. This was carried out over the course of two days, when the weather condition was bright and clear with no rain and wind. Table 1 shows the duration of the re-enactment, categorised by use cases/scenarios. In total, eight different scenarios were re-enacted. In order to facilitate comprehension, we will now proceed to describe in detail the progression of one of the scenarios.

**Vandalism—Group committing graffiti**

**Figure 5:**
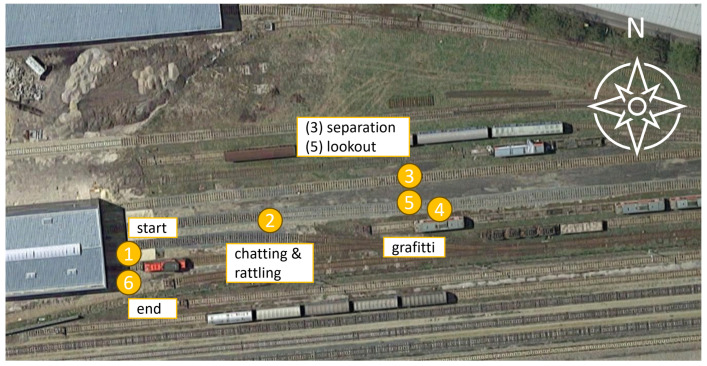
Progression of a scenario in the example group committing graffiti.

Figure 5 shows the progression of the scenario—group committing graffiti.Two people (a lookout and a sprayer) start with the intention of committing vandalism, in the form of graffiti on a train, starting from (1). Both carry spray cans and start walking silently towards (2). When they are about halfway to the target train (4), they start chatting silently and prepare the spray can by shaking it and making rattling noises at position (2). Continuing to chat and rattle, they move towards the train until they reach position (3). At this point, one person moves to start the graffiti at position (4). At the same time, the second person moves to position (5) to act as a lookout for possible disturbances. After some time, the lookout is told by a fictitious security guard to report any interference. As a result, both people try to escape towards position (6), completing the scenario. This scenario takes approximately 2 min.

#### 3.3.2. Data Recording

In all scenarios, the observations made by the detectors were recorded. This included the sensor observations, the fused observations and the ground truth observations. One crucial aspect of utilising the fusion model is the synchronisation of observations. In this study, all of the processing units of the sensors were connected to the same local network. This network included a NTP server, to which all of the processing units responsible for the assignment of timestamps connected. This procedure was also applied for the collection of the ground truth.

Sensor Observation: All sensors provided observations while monitoring the region of interest. Table 1 provides an overview of all the recorded sensor observations. A sensor observation comprises the location (i.e., geo-location) of the detected object, a timestamp, the confidence level and a label. The labels assigned by the video detector consist solely of “person”, indicating that the person detector was based on thermal images. The labels assigned by the acoustic sensors were as follows: speech, rattle and vandalism (e.g., glass breakage). No classification was performed by the radar detector. Consequently, the labels were designated as unknown. It should be noted that the primary objective of the radar detector was to detect movement. Therefore, the sensor observations were utilised as complementary observations for individuals in motion. These data represent the requisite input for the fusion model.

Fusion Observation: One of the principal objectives when utilising our fusion model is to select an appropriate configuration for a specific use case. This necessitates that, during the configuration of the fusion model, we must determine which observations and labels are actually incorporated into the fusion model. This is because the purpose for which the fusion model is intended to be used is of paramount importance. To illustrate, if the objective is to detect graffiti on trains perpetrated by an individual, we consider person detection and movement (thermal and radar sensors) and the rattling of cans (audio sensor) as complementary sensor observations. Consequently, a fused observation is contingent upon the selected configuration and the model parameters (τ, κ). Analogous to the sensor observation, a fusion observation also encompasses the location and the timestamp. The confidence is derived from Equation (8) if the threshold value of κ is exceeded. The label is derived from the configuration used. For example, the label may be vandalism, trespassing or an object lying on the track.

Ground Truth Observations: To determine the performance of the overall system, it is essential to collect the ground truth. The ground truth consists of the time and location of the actors who enacted the scenarios of the use cases. Mobile phones were used for this purpose. An app (https://gpslogger.app/, accessed on 8 May 2024) was installed that recorded the coordinates and timestamps of the actors during the scenarios. This ensured that a ground truth in the form of location and time was measured at all times during the scenarios. It should be noted that determining the GNSS coordinate is also a measurement procedure and is therefore subject to measurement errors (±5 m). This was taken into account during the evaluation. For this reason, the geo-location of the ground truth was not a point but a circle with a radius of 5 m. The ground truth was recorded in the same format as the sensor observations. In Table 1, the total number of ground truth, sensor and fused observations collected is shown. In Figure 1, a schematic example of the geo-location sensor and ground truth observation is depicted.

### 3.4. Evaluation Methodology

The evaluation of (fused) sensor observations is difficult because there is no clear one-to-one correspondence as is common in statistical classification. In a conceptual sense, a sensor observation is considered to be correctly predicted if it coincides with a ground truth observation in both space and time. However, there is a potential issue. A sensor observation can cover several ground truths, for instance, if several people are present, and the sensor observation is imprecise enough to cover all of them. Similarly, a ground truth can be observed by multiple sensors.

Upon examination of a confusion matrix, it becomes evident that the entries for false positives and false negatives are relatively straightforward. A false positive is defined as an observation that lacks an associated ground truth, whereas a false negative is an unobserved ground truth.

A true negative is more challenging to ascertain. Is the absence of observations during the absence of people to be considered a true negative? In essence, this has been the case for the majority of history, given that the temporal scope under consideration is relatively limited. Should every second of that period be regarded as a true negative, which would significantly bias the metrics, or should it be considered as only one true negative? During the course of our trials, we were able to maintain a ground truth at all times, which means that we cannot claim to have observed any true negatives.

**Figure 6:**
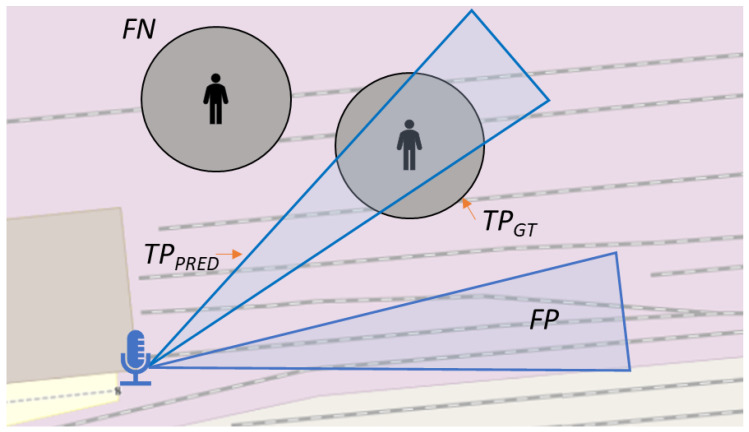
Illustration of the reasoning of the evaluation methodology. The blue polygons represent the area of a sensor observation at a specific time. Grey circles represent the area of the ground truth.

The most significant challenge arises when attempting to identify true positives, as the methodology for counting them is not straightforward. Should every observed ground truth be considered a true positive, or should we also include every sensor observation that correctly identified something? To address this issue, we have developed an alternative approach, which involves disregarding true negatives and categorising true positives into two distinct groups. In Figure 6, the reasoning of the evaluation methodology is illustrated. Since the system deals with geo-localized sensor observations, both the location and the time of the observations must be taken into account. The validity of an event is based on the frequency of the observation. E.g., a sensor observation is valid for 0.5 s if the observation frequency is 2 Hz; vice versa, a ground truth might be valid for 5 s if the frequency of the GPS signal is 0.2 Hz. Thus, we can characterize the classes of the confusion matrix as follows:

- True Positive GT ($TP_{GT}$): This category encompasses ground truths that are spatially and temporally coincident with a sensor observation.
- True Positive PRED ($TP_{PRED}$): This is assigned to sensor observations that align both spatially and temporally with any ground truth.
- False Positive (FP): A sensor observation that does not align with any ground truth is considered a false positive.
- False Negative (FN): A ground truth is counted as a false negative if there is no corresponding sensor observation.

This changes the calculation of the standard metrics as well:

$$\begin{array}{cccc} \; & \mathrm{Accuracy}: & \; & \frac{TP_{GT}+TP_{PRED}}{TP_{GT}+FN+TP_{PRED}+FP}\\ \; & \mathrm{Precision}: & \; & \frac{TP_{PRED}}{TP_{PRED}+FP}\\ \; & \mathrm{Recall}: & \; & \frac{TP_{GT}}{TP_{GT}+FN}\\ \; & F1-\mathrm{Score}: & \; & \frac{2*Precision*Recall}{Precision+Recall}\\ \; & \mathrm{False}\;\mathrm{Positive}\;\mathrm{Rate}\;(\mathrm{FPR}): & \; & 1-Precision \end{array}$$

This leaves the intuitive interpretation of the metrics the same. Accuracy is still the number of correct events divided by the total number of events, and precision is still the number of true predictions divided by the total number of predictions, etc.

For the evaluation, we used the following approach. First, the sensor observations (thermal, acoustic, radar) were evaluated without the fusion component in order to determine the performance of the system with sensors only. This means that the collected sensor observations from the acoustic, thermal and radar detectors were evaluated against the ground truth. We then ran single sensor fusion where we only used one sensor type to see the effect of aggregating and smoothing that is inherent to our fusion model on the results. Subsequently, we ran fusion configurations for different combinations of sensors to identify the best sensor array for each specific use case. At this point, we noted that the evaluation was restricted to the region of interest (ROI) depicted in Figure 4. Consequently, all observations outside of this region were disregarded. This was due to the fact that all of the scenarios were re-enacted within the ROI. Nevertheless, outside of it, there was movement due to pedestrians or cars, which were correctly identified by some of the sensors. However, this would have resulted in a false positive, as no ground truth was available.

## 4. Results

Table 2 shows a comparison of the selected metrics of sensors vs. single sensor fusion categorized by the use cases. We observed an improvement in all of the metrics. For example, when comparing Radar with Radar-Fusion in the use case sabotage, this means the accuracy increased from 39.09% to 53.91%, the F1-score increased from 45.27% to 55.79%, while simultaneously, the FPR was reduced from 61.66% to 46.17%. This behaviour was observed by all other use cases and for thermal as well. In contrast to the observations made in the previous use case, in which acoustic sensors were deployed to detect trespassing and vandalism, a reduction in the metrics was observed in this instance. This phenomenon is discussed in greater detail in the relevant section.

Table 3 shows different fusion combinations categorized by use cases. The primary objective of this comparison was to identify sensor modalities that, when employed in conjunction with one another, yield the most optimal output based on a selected metric. For example, if the recall was chosen as a measure for optimal performance, for the use case sabotage, either the fusion of all sensors (All-Sensor Fusion) or the fusion of only thermal and radar sensor would yield the best output with 73.09%. Looking at the use case trespassing, the fusion of acoustic and thermal, with 87.91%, resulted in the best performance. Respectively, for the use case vandalism, the fusion of all three sensors achieved the best performance with 82.96%. This procedure can be conducted with any of the metrics. In fact, Table 3 shows that selecting one of the other three metrics (Accuracy, FPR or F1 Score), the fusion of acoustic and thermal exclusively yielded the best performance. Nevertheless, the results of the evaluation support the assumption that suitable fusion configurations need to be found based on a selected performance metric for each use case. The selection of the metric is therefore crucial and should always be chosen based on the application requirements. To illustrate, if the operators of the railway security system aim to minimise the number of false alarms while maintaining a high level of detection accuracy for the trespassing use case, it would be advisable to configure our fusion model to fuse only acoustic and thermal observations.

## 5. Discussion

Table 2 illustrates the efficacy of our fusion model in enhancing the accuracy and F1-score, while reducing false alarms, when applied to observations generated by thermal and radar sensors. This is evident across all use cases. Our analysis has revealed that this phenomenon occurs when a sensor is generating a substantial number of observations, including instances of false positives. The inference system employed by our model enables it to only trigger an alarm when sufficient information is present over time. Outliers that are characterised with less confidence will not trigger an alarm because the threshold described in Equation (9) will not be exceeded. This results in better accuracy and less false alarms. On the one hand, this behaviour shows high potential, for outliers, but there is a downside of this mechanism as well. In Table 2, we see that for the acoustic fusion, the accuracy and reduction in false alarms did, in fact, deteriorate. The acoustic sensor observations exhibited a lower degree of confidence compared to the other sensors. Consequently, the utilisation of the identical configuration (as employed for radar and thermal) did not yield as many fused observations. This can be retraced to the selection of the threshold in (9) and configuration of the fusion model. Nevertheless, the results demonstrate that identifying an optimal configuration for the fusion model is crucial for achieving optimal performance. We believe that even when using a single sensor modality as mentioned in [5,6,7,8], an improvement can be shown by finding a good configuration for our fusion model, which differs on a use case basis.

The full potential of our fusion model is, however, revealed when multiple sensor modalities are combined. The results of this work clearly support the idea that fusing everything together will not yield the best performance (based on a selected metric). It is therefore crucial to analyse the sensor data in terms of complementarity when configuring our fusion model. It is necessary to find out which of the sensor data is complementary, redundant or even contradictory. This knowledge allows the appropriate fusion configuration to be selected. In our work, we have performed this by comparing the results of different fusion configurations regarding their achieved performance. Table 3 shows that it is not possible to select only one configuration for all of the use cases. This is because the described use cases comprise certain characteristics (movement, speech, rattling, etc.). These characteristics determine the optimal configuration set that facilitates the use-case-specific integration of complementary sensor data (e.g., thermal and acoustic for the detection of a person walking and chatting). This phenomenon has been observed in our work since the configuration sets yielding the best performance (in comparison to the system without the fusion model) differ in the use cases. For instance, our evaluation indicates that the optimal performance for the trespassing use case is achieved by fusing acoustic and thermal sensor observations, provided that accuracy or a false positive rate are the prioritised performance metrics. Conversely, the fusion of all three sensor observations yields the optimal performance for the vandalism use case when recall is selected as the performance metric.

Upon examination of the absolute values of the evaluated metrics, it becomes evident that they do not meet the expectations of an operator in real-world scenarios. As previously stated in the introduction, the authors of [6] describe the reduction in the false positive rate (FPR) to 0.24% for a trespassing use case. In our work, the lowest FPR in the use case of trespassing was achieved by the fusion of acoustic and thermal observations (9.71%). This is due to the fact that the sensors used in this work were not optimised (in terms of calibration and detectors) for the specific use cases. However, we believe that our fusion model offers a reinforcement effect on the quality of the input data. This implies that an improvement in the quality of the sensor data will also result in an enhancement of the output performance of our fusion model. This phenomenon is corroborated by the findings presented in Table 2 and Table 3.

Nevertheless, for all use cases, we can demonstrate that by applying our fusion model, we have successfully increased accuracy and F1-score while reducing the relative number of false alarms. In the cited work [12], the authors describe a methodology based on weighted maps, which is comparable to our approach. Although the use cases differ, the authors of the aforementioned paper argue that they can reduce the number of false alarms while maintaining the detection accuracy. Our model, however, demonstrates that it is possible to reduce the number of false alarms while simultaneously increasing the accuracy and F1-score.

In this work, we also identified potential shortcomings of our fusion model. The core of the inference system utilises Bayes’ Theorem in a log-odds form (see Equation (4)). For updating a cell in a POM, the likelihood of a cell, given a sensor observation, is required. In this work, we assume that this is modelled by the sensors, resulting in a confidence that is inferred in our fusion model. It is important to note that our approach lacks any means of verification regarding the validity of the information it provides. For instance, it is possible that an acoustic sensor may correctly detect an event, but incorrectly estimate the angle of arrival due to a very loud surrounding. Consequently, the sensor may report observations with high confidence, even though they are false alarms with respect to the event’s position. A similar example is that a radar sensor cannot distinguish between the movement of people and, for instance, that of animals (rabbits, cats, etc.). In fact, when evaluating the data, such a situation occurred. Nevertheless, in such a scenario, a sensor reports observations with high confidence, which are considered false alarms in the related use case. Since our fusion model does not incorporate this information, we consider it to be valid. As a result of the sensors providing consistent positive feedback, sufficient information will be available over time to trigger a fused observation, which is considered a false alarm.

The evaluation reveals that identifying the optimal configuration is a challenging process. As previously stated, the optimal configuration is contingent upon the selected metric and is specific to a given use case. In theory, it is possible to define an optimization process that minimizes a cost function based on a selected metric. This approach enables the identification of an optimal configuration set, in terms of the cost function, that yields optimal performance for each use case. It is our contention that such configuration sets can be found. This approach will be the subject of further investigation in future work. Furthermore, it is our strong belief that this approach can also be used in other sensing applications, such as search and rescue. In our future work, we intend to investigate the applicability of our approach in this field.

## 6. Conclusions

In this work, we presented a Bayesian approach for the purpose of robust detection of critical events in the context of railway security. The proposed fusion model was evaluated on a dataset of eight scenarios covering the use cases sabotage, trespassing and vandalism. The scenarios were enacted at a railway depot in Austria covering an area of roughly (30 m × 150 m). We analysed the effectiveness of different sensor fusion configurations in different use cases, with the aim of improving accuracy and minimising false alarms. The results showed that sensor fusion can improve performance when configured correctly, although the optimal combination of sensors varies depending on the use case and the metric chosen. Here are the key takeaways from our research:

- Performance varies by sensor type and use case: We found that each type of sensor had unique strengths and weaknesses in different use cases. For example, the acoustic sensor showed better recall in detecting glass breakage and graffiti, while the radar sensor performed better in detecting other activities. Our fusion model allowed us to improve accuracy and F1 score while simultaneously reducing false alarms.
- Fusion configurations must be tailored to use cases: Our results emphasised the importance of tailoring sensor fusion configurations to specific use cases. The optimal fusion setup varied depending on the desired metric, such as recall, accuracy or FPR. For example, for the trespassing use case, the combination of acoustic and thermal sensors yielded the best performance, while for vandalism, the fusion of all three sensor modalities was most effective.
- Challenges with sensor data quality and fusion configurations: Despite the improved performance of sensor fusion, there were limitations due to the quality of the sensor data and the configurations chosen. For example, some sensors could report false alarms due to misinterpretation of noise or other activities. The inference system’s reliance on sensor confidence could lead to incorrect outputs if sensors reported inaccurate information with high confidence.
- Potential for further optimization and future work: We identified the need for further optimisation to find the best configuration for each use case. A more structured approach, such as using a cost function to optimise configurations, could help to address this challenge. In addition, we believe that our fusion model could be applied to other sensing applications, such as search and rescue, to improve detection and reduce false alarms.

Overall, this research demonstrated the potential benefits of sensor fusion in improving detection accuracy and reducing false alarms in various use cases. However, it also highlighted the need for careful configuration and optimisation to ensure the best results. Future work will focus on refining the fusion model, exploring other sensor applications and improving sensor data quality to improve overall system performance.

## Figures and Tables

**Table 1:** Summary of the total duration of re-enacted scenarios categorized by the use cases.

Use Case/Scenario	Duration	Number of Observations			
	[HH:MM:SS]	Acoustic	Radar	Thermal	gt
**U1—sabotage**	**0:22:00**	**152**	**13,630**	**45,023**	**650**
wantonly derailing	0:06:51	8	4442	10,761	451
backpack on trail	0:15:09	144	9188	34,262	199
**U2—trespassing**	**0:22:39**	**252**	**13,570**	**39,682**	**479**
person lying on track	0:08:00	4	3713	7862	268
person crossing track	0:05:34	58	5663	6728	87
group crossing tracks	0:09:05	190	4194	25,092	124
**U3—vandalism**	**0:27:48**	**804**	**10,891**	**43,767**	**722**
person committing graffiti	0:10:50	327	3799	11,468	118
group committing graffiti	0:11:28	477	4840	25,636	295
**breaking window**	0:05:30	0	**2252**	**6663**	309
**Gesamtergebnis**	**1:12:26**	**1208**	**38,091**	**128,472**	**1851**

**Table 2:** Evaluation results for sensors and single sensor fusion.

	Acoustic	Acoustic-Fusion	Radar	Radar-Fusion	Thermal	Thermal-Fusion
**Accuracy [%]**						
U1—sabotage	25.23%	31.86%	39.09%	53.91%	62.51%	69.63%
U2—trespassing	39.42%	37.35%	46.13%	64.90%	83.47%	90.19%
U3—vandalism	61.79%	59.43%	45.30%	64.86%	74.03%	78.77%
**FPR [%]**						
U1—sabotage	29.01%	13.26%	61.66%	46.17%	37.52%	30.31%
U2—trespassing	34.35%	39.46%	54.25%	34.91%	16.48%	9.59%
U3—vandalism	36.32%	38.03%	55.38%	34.44%	25.88%	20.90%
**Recall [%]**						
U1—sabotage	19.08%	22.67%	57.29%	58.76%	61.98%	63.72%
U2—trespassing	32.81%	30.33%	68.99%	68.66%	77.84%	79.07%
U3—vandalism	68.69%	66.07%	57.30%	55.91%	66.76%	66.88%
**F1 Score [%]**						
U1—sabotage	28.92%	33.49%	45.27%	55.79%	61.62%	65.35%
U2—trespassing	40.70%	38.84%	52.20%	64.20%	79.38%	83.24%
U3—vandalism	62.56%	60.19%	49.47%	58.36%	69.44%	71.76%

**Table 3:** Evaluation results of the fusion of different sensor combinations.

	Acoustic-Radar-Fusion	All-Sensor-Fusion	Acoustic-Thermal-Fusion	Thermal-Radar-Fusion
**Accuracy [%]**				
U1—sabotage	53.93%	60.87%	69.67%	60.87%
U2—trespassing	65.33%	72.89%	90.22%	72.89%
U3—vandalism	64.43%	73.64%	77.75%	74.62%
**FPR [%]**				
U1—sabotage	46.18%	39.24%	30.32%	39.24%
U2—trespassing	34.68%	27.14%	9.71%	27.14%
U3—vandalism	35.36%	26.40%	22.32%	25.31%
**Recall [%]**				
U1—sabotage	59.51%	73.09%	66.76%	73.09%
U2—trespassing	73.61%	84.98%	87.91%	84.98%
U3—vandalism	68.34%	82.96%	81.01%	76.45%
**F1 Score [%]**				
U1—sabotage	56.15%	65.56%	67.51%	65.56%
U2—trespassing	67.40%	76.39%	88.65%	76.39%
U3—vandalism	64.09%	77.64%	78.53%	75.15%

## Data Availability

The data that support the findings of this study are part of a research project and are not publicly available.

## Abbreviations

The following abbreviations are used in this manuscript:

CCTV	Closed Circuit Television
DNN	Deep Nural Network
FOV	Field OF View
GIS	Geographical Information System
GPU	Graphics Processing Unit
GT	Ground Truth
POM	Probabilistic Occupancy Map
R-CNN	Region Based Convolutional Neural Network
ROI	Region Of Interest
NTP	Network Time Protocol
